# An analysis of preclinical efficacy testing of antivenoms for sub-Saharan Africa: Inadequate independent scrutiny and poor-quality reporting are barriers to improving snakebite treatment and management

**DOI:** 10.1371/journal.pntd.0008579

**Published:** 2020-08-20

**Authors:** Stuart Ainsworth, Stefanie K. Menzies, Nicholas R. Casewell, Robert A. Harrison

**Affiliations:** Centre for Snakebite Research and Interventions, Liverpool School of Tropical Medicine, Pembroke Place, Liverpool, United Kingdom; Instituto de Biomedicina de Valencia, SPAIN

## Abstract

**Background:**

The World Health Organization’s strategy to halve snakebite mortality and morbidity by 2030 includes an emphasis on a risk-benefit process assessing the preclinical efficacy of antivenoms manufactured for sub-Saharan Africa. To assist this process, we systematically collected, standardised and analysed all publicly available data on the preclinical efficacy of antivenoms designed for sub-Saharan Africa.

**Methodology/Principal findings:**

Using a systematic search of publication databases, we focused on publicly available preclinical reports of the efficacy of 16 antivenom products available in sub Saharan Africa. Publications since 1999 reporting the industry standard intravenous pre-incubation method of murine *in vivo* neutralisation of venom lethality (median effective dose [ED_50_]) were included. Eighteen publications met the criteria. To permit comparison of the several different reported ED_50_ values, it was necessary to standardise these to microlitre of antivenom resulting in 50% survival of mice challenged per milligram of venom (μl/mg). We were unable to identify publicly available preclinical data on four antivenoms, whilst data for six polyspecific antivenoms were restricted to a small number of venoms. Only four antivenoms were tested against a wide range of venoms. Examination of these studies for the reporting of key metrics required for interpreting antivenom ED_50_s were highly variable, as evidenced by eight different units being used for the described ED_50_ values.

**Conclusions/Significance:**

There is a disturbing lack of (i) preclinical efficacy testing of antivenom for sub Saharan Africa, (ii) publicly available reports and (iii) independent scrutiny of this medically important data. Where reports do exist, the methods and metrics used are highly variable. This prevents comprehensive meta-analysis of antivenom preclinical efficacy, and severely reduces the utility of antivenom ED_50_ results in the decision making of physicians treating patients and of national and international health agencies. Here, we propose the use of a standardised result reporting checklist to resolve this issue. Implementation of these straightforward steps will deliver uniform evaluation of products across laboratories, facilitate meta-analyses, and contribute vital information for designing the clinical trials needed to achieve the WHO target of halving snakebite morbidity and mortality by 2030.

## Author summary

Antivenom is the first-choice therapy for victims of snakebite envenoming. Currently there is very little robust evidence that many of the antivenoms currently being used in Africa are suitable or effective. Unusually for a human medicine, clinical trials are not a pre-requisite for antivenom approval, licensing and use in patients. This leaves a situation where nearly all the information of an antivenom’s effectiveness is based on mouse assays assessing neutralisation of venom-induced lethality, so-called “preclinical” antivenom testing. Here we analysed all the publicly available preclinical data on antivenoms for Africa published in the last 20 years. Our results demonstrate that there is worryingly little publicly available information on the preclinical efficacy of antivenoms and that the efficacy of some products is seemingly very weak. We hope that the World Health Organization’s antivenom assessment and listing scheme (independently and systematically testing antivenom efficacy and quality) will fill many of these crucial information gaps. Furthermore, the quality of the result reporting was highly variable across studies, making meaningful comparisons difficult and causing challenges for clinicians treating snakebites to rapidly decipher efficacy information. To remedy this, we have developed a reporting checklist to harmonise preclinical antivenom efficacy reporting across the globe.

## Introduction

Snakebite envenoming (SBE) is a Neglected Tropical Disease that annually kills 85,000–130,000 and maims >400,000 people living in the world’s most disadvantaged communities [1,2]. The lack of safe, effective antivenoms in many parts of the tropics is the main driver of the continuing high mortality and morbidity rates observed in these regions. The World Health Organization (WHO) has identified the supply of safe and effective treatments as one of four key objectives to halve SBE mortality and morbidity by 2030 [3].

Antivenom (purified polyclonal immunoglobulin from venom-immunised animals) is the first-choice therapy for SBE. Due to the method of manufacture, antivenom effectiveness is largely restricted to the venom/s used for animal immunisation [4,5]. Differences in venom composition can vary substantially between species, and even between different locales of the same species [6–8]. The geographic origin of venom immunogens can therefore influence the geographic effectiveness of antivenoms [9].

Antivenoms are atypical therapeutics since they have often been deployed for human use without undergoing traditional Phase 1, 2 and 3 clinical testing [10]. A recent review of the clinical effectiveness of antivenoms for sub-Saharan Africa identified that only three of the antivenoms currently used in Africa are supported by robust clinical trial data [11]. In the absence of robust clinical trials, the small number of good-quality clinical reports (primarily prospective observational cohort studies or retrospective analyses) suggest the effectiveness of many products is limited. As further observed by Potet et al, no robust and reliable clinical information is available for seven antivenoms currently in use in sub-Saharan Africa [11].

Thus in many countries, in the absence of clinical data, the preclinical neutralisation of venom lethality in a mouse model is seemingly the only antivenom efficacy data required before a product is registered and used clinically [10]. Due to the absence or paucity of independently generated preclinical information for many antivenoms, it is often the manufacturer’s results of preclinical efficacy, which are very rarely published, that form the only basis of decision making. This means antivenoms are often deployed without robust, independently-scrutinised, preclinical or clinical evidence of their efficacy or effectiveness—sometimes with fatal consequences [11–14]. A tragic 2008 example of this was the reported increase in mortality of antivenom-treated patients from 1.8% to 12.1% following the introduction into West Africa of an antivenom manufactured with venom from Indian snakes [12]. Recent publications indicate that this is still not uncommon [13,14]. Consequently, efforts are urgently required to better assist and simplify the decision making of physicians treating snakebite patients, and of government and other medicine-procuring agencies [3].

The WHO recommends that preclinical testing of antivenoms follows a two-step process; a preliminary *in vitro* assessment followed by essential *in vivo* preclinical analyses [10]. The preliminary *in vitro* approach (e.g. using methods such as ELISA, immunoblotting [15] or antivenomics [16]) is primarily to identify antivenoms which are likely to lack efficacy during essential *in vivo* assays, and are thus crucial to reducing the number of animals required for testing.

Following adequate preliminary *in vitro* assessment, antivenoms must be assessed via the standard assay of preclinical efficacy testing; the *in vivo* murine antivenom “neutralisation of venom lethality” or “median effective dose (ED_50_)” assay (for the original description of the assay see [17] and for a detailed review of the assay see [18,19]). The assay is performed by mixing a fixed dose of venom that induces death in 100% of mice (usually 2.5–5.0 fold the median lethal dose [LD_50_]–the amount of venom that kills 50% of injected mice) with varying volumes of antivenom, and intravenously (IV–although some practitioners use the intraperitoneal route) injecting this venom/antivenom mixture into mice following a 30 minute incubation step at 37 ^o^C. Survival of injected mice after a specified time interval (usually 24 hours) are recorded and data analysis is used to calculate the ED_50_—the dose of antivenom that results in survival of 50% of injected mice.

Increasing the availability and transparency of available information on antivenom preclinical efficacy can contribute to validating manufacturer claims, provide independent assessment of efficacy, and aid regulatory bodies and health care providers to make informed choices. This is reflected by the fact that the WHO, prior to a stock-piling initiative, is currently undertaking an assessment and listing of antivenoms for sub-Saharan Africa [3]. It is hoped that such an assessment will identify products with favourable “risk‒benefit ratios”, and thus aid health agencies and other relevant stakeholders to make informed decisions on antivenom procurement. We hope this valuable evidence will be made available for scrutiny by the wider SBE community–in particular to those designing clinical trials.

However, lack of international consensus in preclinical antivenom testing has the potential to threaten this approach. Inadequate and non-standardised reporting of the results of animal research is a major contributor to poor experimental reproducibility and prevents harmonisation of results for meta-analyses. Addressing these issues for murine antivenom-efficacy experiments will help provide the evidence needed to guide clinical trial and clinician decision making. In this context, the 2011 ARRIVE (Animal Research: Reporting of *In Vivo* Experiments) guidelines provide protocols to improve animal reporting and experimental reproducibility [20]. Since publication, the ARRIVE guidelines have been endorsed by >1000 international journals, research institutes and funders [21] but their uptake by individual research groups and enforcement by many journals has been relatively poor [22].

Here, using the ARRIVE guidelines as a basis for comparison, we assess the extent to which current reporting of preclinical antivenom efficacy results across studies presents the information required to allow thorough scrutiny and understanding of results presented, and how adoption of standardised reporting of results will increase the value of data that can be obtained. Furthermore, we aim to provide information that will assist researchers, clinicians and health agencies, and to identify areas in which future preclinical antivenom research needs to be focused and those that need urgent improvement in the interim. To that end, we investigated all ED_50_ antivenom-efficacy publications of snakebite therapeutics used in sub-Saharan Africa in the past 20 years.

## Methods

### Definitions

Several key terms are used throughout this manuscript which we wish to define to aid comprehension.

*Median lethal dose* (LD_50_): the quantity of snake venom that leads to the death of 50% of the animals in a group after an established period of time [10].

*Median effective dose* (ED_50_): the quantity (expressed as a ratio of μl antivenom per mg of venom) of antivenom that protects 50% of test animals injected with a lethal dose (typically 2.5–5 x LD_50_) of venom [10]. Alternatively, this can be viewed as the quantity of antivenom that neutralises all but 1 x LD_50_.

*Efficacy*: the efficacy of an antivenom is a measure of the *in vivo* or *in vitro* neutralising potency against a specific activity of a venom or venoms [10].

*Effectiveness*: the effectiveness of an antivenom is a measure of its ability to produce a clinically effective outcome when used to treat snakebite envenoming [10].

*Monospecific/monovalent antivenom*: Raised from the venom of a single species and are limited in use to that species or to a few closely related species.

*Polyspecific/polyvalent antivenom*: Raised from a mixture of venoms from more than one species of venomous snake.

### Antivenoms

We focussed our assessment on the same 16 antivenoms that featured in the valuable recent assessment of the clinical effectiveness and safety of antivenom treatment of snakebite victims in sub-Saharan Africa [11] (Table 1). We recorded details on the preclinical efficacy of each antivenom using data from product packaging or inserts provided by each manufacturer.

**Table 1:** Commercially available, or recently available, African antivenom products. This table is based on estimation of available antivenom products by Potet et al. 2019 [11]. * = manufacturing currently on hold.

Antivenom brand name	Manufacturer	Country of production	Species venoms neutralized according to product insert	Animal Source	Type of Ig fragment
Antivipmyn-Africa	Instituto Bioclon (Silanes)	Mexico	*B*. *arietans*, *B*. *gabonica*, *B*. *rhinoceros*, *D*. *angusticeps*, *D*. *jamesoni*, *D*. *polylepis*, *D*. *viridis*, *E*. *leucogaster*, *E*. *ocellatus*, *E*. *pyramidum*, *N*. *haje*, *N*. *katiensis*, *N*. *melanoleuca*, *N*. *nigricollis*, *N*. *nivea*	Equine	F(ab’)_2_
ASNA antivenom C	Bharat Serum and Vaccines	India	*B*. *arietans*, *B*. *gabonica*, *B*. *nasicornis*, *D*. *angusticeps*, *D*. *jamesoni*, *D*. *polylepis*, *E*. *carinatus*, *N*. *haje*, *N*. *melanoleuca*, *N*. *nigricollis N*. *nivea*	Equine	F(ab’)_2_
ASNA antivenom D (ASNA-D)	Bharat Serum and Vaccines	India	*B*. *arietans*, *B*. *gabonica*, *B*. *nasicornis*, *D*. *angusticeps*, *D*. *jamesoni*, *D*. *polylepis*, *E*. *ocellatus*, *N*. *haje*, *N*. *melanoleuca*, *N*. *nigricollis*, *N*. *nivea*	Equine	F(ab’)_2_
EchiTAbG	MicroPharm	United Kingdom	*E*. *ocellatus*	Ovine	IgG
EchiTAb-Plus-ICP	Instituto Clodomiro Picado	Costa Rica	*B*. *arietans*, *E*. *ocellatus*, *N*. *nigricollis*	Equine	IgG
Fav-Afrique*	Sanofi-Pasteur	France	*B*. *arietans*, *B*. *gabonica*, *D*. *jamesoni*, *D*. *polylepis*, *D*. *viridis*, *E*. *leucogaster*, *E*. *ocellatus*, *N*. *haje*, *N*. *melanoleuca N*. *nigricollis*	Equine	F(ab’)_2_
Inoserp Pan Africa	Inosan	Mexico/ Spain	*B*. *arietans*, *B*. *gabonica*, *B*. *rhinoceros*, *D*. *angusticeps*, *D*. *jamesoni*, *D*. *polylepis*, *D*. *viridis*, *E*. *leucogaster*, *E*. *ocellatus*, *E*. *pyramidum*, *N*. *haje*, *N*. *katiensis*, *N*. *melanoleuca*, *N*. *nigricollis*, *N*. *nivea*, *N*. *pallida*	Equine	F(ab’)_2_
Snake Venom Antiserum (Pan Africa)	Premium Serums	India	*B*. *arietans*, *B*. *gabonica*, *B*. *nasicornis*, *B*. *rhinoceros*, *D*. *angusticeps*, *D*. *jamesoni*, *D*. *polylepis*, *D*. *viridis*, *E*. *carinatus*, *E*. *leucogaster*, *E*. *ocellatus*, *N*. *nigricollis*, *N*. *haje*, *N*. *melanoleuca*	Equine	F(ab’)_2_
Snake Venom Antiserum (Central Africa)	Premium Serums	India	*B*. *rhinoceros*, *E*. *carinatus*, *D*. *russelli*, *D*. *polylepis*	Equine	F(ab’)_2_
SAIMR Boomslang	South African Vaccine Producers	South Africa	*D*. *typus*	Equine	F(ab’)_2_
SAIMR Echis	South African Vaccine Producers	South Africa	*E*. *carinatus*, *E*. *ocellatus*, *E*. *coloratus*, *Cerastes* spp.	Equine	F(ab’)_2_
SAIMR Polyvalent	South African Vaccine Producers	South Africa	*B*. *arietans*, *B*. *gabonica*, *D*. *angusticeps*, *D*. *jamesoni*, *D*. *polylepis*, *H*. *haemachatus*, *N*. *annulifera*, *N*. *melanoleuca*, *N*. *mossambica*, *N*. *nivea*	Equine	F(ab’)_2_
Snake Venom Antiserum Polyvalent (equine)	VACSERA	Egypt	*B*. *arietans*, *B*. *gabonica*, *C*. *cerastes*, *C*. *vipera*, *E*. *carinatus*, *E*.*coloratus*, *M*. *lebetina*, *M*. *palestinae*, *N*. *haje*, *N*. *melanoleuca*, *N*. *mossambica*, *N*. *nigricollis*, *N*. *oxiana*, *P*. *persicus*,*V*. *ammodytes*, *V*. *xanthina*, *W*. *aegyptia*	Equine	F(ab’)_2_
VINS Snake Venom Antiserum (Central Africa)	VINS Bioproducts	India	*B*. *gabonica*, *E*. *carinatus*, *D*. *russelli*, *D*. *polylepis*	Equine	F(ab’)_2_
VINS Snake Venom Antiserum (Echis)	VINS Bioproducts	India	*E*. *ocellatus*	Equine	F(ab’)_2_
VINS Snake Venom Antiserum (Pan Africa)	VINS Bioproducts	India	*B*. *arietans*, *B*. *gabonica*, *D*. *jamesoni*, *D*. *polylepis*, *D*. *viridis*, *E*. *leucogaster*, *E*. *ocellatus*, *N*. *haje*, *N*. *melanoleuca*, *N*. *nigricollis*	Equine	F(ab’)_2_

### Publication search strategy

We searched for publications relating to any of the 16 antivenom products listed in Table 1 on PubMed, Web of Science and Scopus. The keywords “Antiven* and Africa” or “Anti-snake* and Africa” or “ASV and Africa” or “Anti-ophidic and Africa”, were used in association with: i) “ED_50_” or ii) “LD_50_” or iii) “Preclinical” or iv) individual product names (a full list of search terms and papers returned can be found in S1 and S2 Files, respectively). As availability of individual antivenom brands in sub-Saharan Africa has changed dramatically within the last twenty years (products being withdrawn and new products entering the market), results were limited to publications since 1999. Only results representing neutralisation of lethality experiments (ED_50_) via the intravenous route and performed in mice were included. Only papers in English, French, Spanish and Portuguese were included. An initial search was performed on 2^nd^ of August 2019 followed by a second search on 11^th^ May 2020 (note, access to Scopus was unavailable on this occasion).

### Standardisation of values and assessment of the quality of reporting

For each publication that met the criteria for inclusion, we recorded whether each study reported the basic details required for interpreting ED_50_s. To do this, we assessed the identified publications against four main criteria adapted from recommendations of the ARRIVE guidelines for *in vivo* reporting [20].

*Experimental set up*: The assessment criteria were: i) Details of venom supplier. ii) Geographical origin of the snake venom(s). iii) Details of antivenom(s) used including source, batch and expiry date. iv) Antivenom total protein content (in mg total protein/ml of antivenom) and the method by which it was determined. v) Ethical compliance statement. vi) Conflict of interest statement.

*Animals*: The assessment criteria were: i) Source of animals. ii) Strain. iii) Weight. iv) Sex. v) Basic husbandry details.

*Procedure*: The assessment criteria were: i) Stating of control groups. ii) Number of animals per group. iii) Total number of animals and number of groups used. iv) Multiples of LD_50_ used to determine ED_50_. v) If and how antivenom was pre-incubated with venom. vi) Route of administration. vii) Injection volume. viii) Experiment duration.

*Result reporting*: The assessment criteria were: i) Mouse group outcome reporting. ii) Presence or absence of adverse events. iii) Description of statistical analysis. iv) Units used to describe ED_50_s.

Due to the various metrics of antivenom efficacy used in these reports, and to enable cross-comparison of the results, all ED_50_ values were standardised to the dose of antivenom, in microlitres, which prevents lethality in 50% of test mice per milligram of venom (μl/mg)–a recommend metric detailed in the WHO guidelines[10]. Calculations used for conversions are available in S3 File. All statistical analyses were performed using GraphPad Prism 5.

## Results

### Summary

The database search returned a total of 505 publications (S2 File). Only 18 publications met our criteria for inclusion: murine studies published since 1999 that described the standard assay of preclinical neutralisation of venom lethality using any of the 16 antivenoms indicated for envenoming by African snake species.

**Fig 1:**
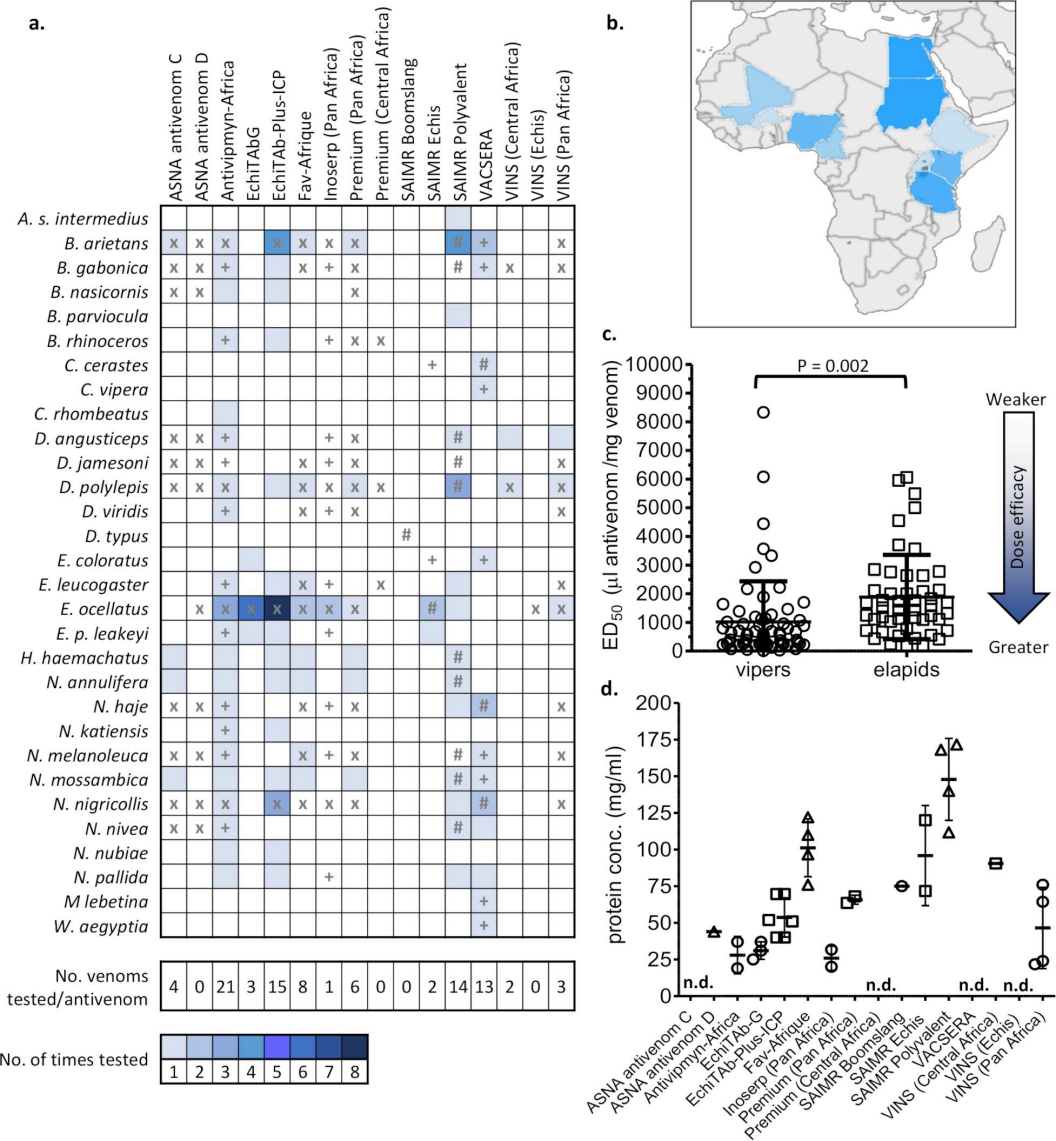
Summary of breadth and frequency of publicly available preclinical testing data of available African antivenoms vs. medically important snakes of the region. **a)** The heat map represents the number of times each antivenom has been tested against a venom. x = antivenom manufacturer makes specific claims of neutralisation against this venom. + = antivenom manufacturer makes specific claims of para-specific neutralisation against this venom (i.e. venom not used as an immunogen). # = manufacturer has claimed this venom is used for immunisation during antivenom manufacture. Antivenom names as in Table 1. **b)** The geographical origin of the snake venoms used in prior preclinical studies of African antivenom efficacy. The darker the blue colour, the more venoms that were investigated from that country. Countries without colour did not have any venoms represented in the preclinical testing in the identified studies. **c)** The preclinical efficacy of African antivenoms against venoms from vipers and elapids (see S1 Table for species classification, individual values and references). ED_50_ values represented as dose of antivenom, in μl, which prevents lethality in 50% of test mice per mg of venom (μl/mg). Significance (P) determined using an unpaired t test with Welch's correction. **d)** Total protein content of African antivenoms in this study. Bold bar represents the mean, whiskers represent standard deviation of the mean. Values and references supporting this data are available in S2 Table. Bars represent standard deviation of the mean, and n.d. indicates not determined/available.

In total, the preclinical efficacy of these African antivenoms against venoms from 29 snake species (Fig 1A) sourced from only 11 different African countries (Fig 1B) were examined. Two venoms were designated as originating from “North” or “East Africa”, whilst the origin of 35 venoms was not stated.

Of the 16 antivenoms available (historically and/or contemporarily) in sub-Saharan Africa, 12 had preclinical data publicly available, and a complete list of the summarised, standardised ED_50_ and potency values for each can be found in S1 Table. Worryingly, we could not find any published evidence for the preclinical efficacy of the ASNA antivenom D (Bharat Serum and Vaccines, India), Premium Snake Venom Antiserum (Central Africa) (Premium Serums, India), VINS Snake Venom Antiserum (Echis) (VINS Bioproducts, India) and SAIMR Boomslang (South African Vaccine Producers, South Africa) antivenoms (although alternative methods of determining preclinical efficacy have been applied to the SAIMR Boomslang product [23]).

There exists little publicly available data relating to the efficacy of the majority of polyspecific antivenoms, with each examined against one to six venoms (Table 1), including VINS Snake Venom Antiserum (Pan Africa) (VINS Bioproducts, India), VINS Snake Venom Antiserum (Central Africa) (VINS Bioproducts, India), Inoserp (Pan Africa) (Inosan, Spain/Mexico), Premium Snake Venom Antiserum Pan Africa (Premium Serums, India) and ASNA antivenom C (Bharat Serum and Vaccines, India), some of which are widely available in sub-Saharan Africa and indicated to treat envenoming by multiple snake species. Conversely, polyspecific antivenoms Antivipmyn-Africa (Instituto Bioclon [Silanes], Mexico), Snake Venom Antiserum Polyvalent (equine) (VACSERA, Egypt), herein referred as “VACSERA”, SAIMR Polyvalent (South African Vaccine Producers, South Africa) and trivalent antivenom EchiTAb-Plus-ICP (Instituto Clodomiro Picado, Costa Rica) were tested against multiple snake species’ venoms (21, 13, 14 and 15 different venoms, respectively) (Table 1). The efficacy data for Antivipmyn-Africa, VACSERA and EchiTAb-Plus-ICP was near exclusively supplied from laboratories associated with the antivenom manufacturers.

Six studies reported antivenoms lacking preclinical efficacy against one or more snake venoms (S1 Table), but the majority of these concerned venoms that were either not used in manufacture (where stated) or for which the antivenoms are not indicated for (e.g., EchiTAb-Plus-ICP vs. *D*. *polylepis*, *N*. *annulifera*, *H*. *haemachatus*, *N*. *katiensis*, *N*. *mossambica* and *N*. *nubiae* [24]; VINS Snake Venom Antiserum (Central Africa) vs. *D*. *angusticeps* [25] and ASNA antivenom C vs. *N*. *annulifera*, *N*. *mossambica* and *H*. *haemachatus* [26]). This emphasises the general species specificity of antivenoms. VINS Snake Venom Antiserum (Pan Africa) was the only antivenom described as lacking efficacy against a venom for which it is indicated for (*E*. *ocellatus* [27]).

**Fig 2:**
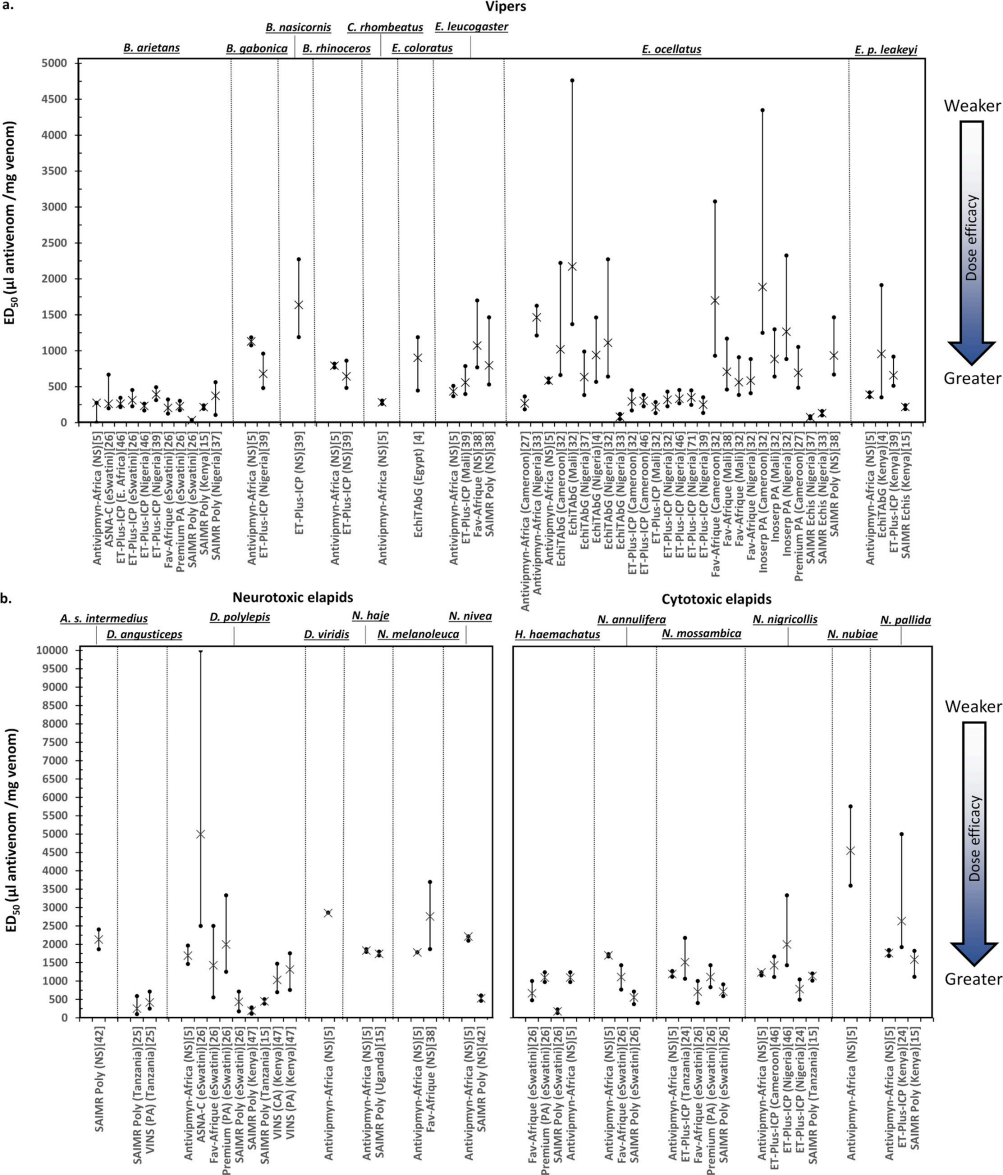
The preclinical efficacy (ED_50_ values) of African antivenoms vs. **a)** viper venoms and **b)** neurotoxic elapid venoms and cytotoxic elapid venoms. Note difference in axis scales. Values represented as dose of antivenom, in μl, which prevents lethality in 50% of test mice per mg of venom (μl/mg). Bars are 95% confidence intervals. Where available, venom origin is named by country. Data reference is in square brackets. All data is additionally listed in S1 Table. Antivenom names are as in Table 1 with the following exceptions: ET-Plus-ICP = EchiTAb-Plus-ICP. PA = Pan Africa. CA = Central Africa. Poly = Polyvalent. NS = not specified.

There was a statistically significant trend (unpaired t-test with Welch’s correction, P = 0.002) of antivenoms possessing more efficacious mean ED_50_ values against viper venoms (n = 67, mean: 1021.0 μl/mg, Std. Deviation [SD]: ±1411 μl/mg, range: 14.90–8333.0 μl/mg) than elapid venoms (n = 49, mean: 1884.0 μl/mg, SD: ±1469.0 μl/mg, range: 175.4–6061.0 μl/mg) indicating that relatively more viper venom is neutralised per ml of antivenom (Fig 1C). It is tempting to speculate that this observation may be the result of viper venoms typically being dominated by higher molecular mass proteins [28] (and thus more immunogenic), than typically low molecular mass dominated (e.g. <14 kDa three finger toxin and phospholipase A_2_ family) elapid venoms. SAIMR Polyvalent is a notable exception to this observation, demonstrating consistently more-efficacious anti-elapid (and more anti-viper-like) ED_50_s, compared to other polyvalent antivenoms (Fig 1C, Fig 2, S1 File). This could be due to the exceptionally high total protein concentration of SAIMR Polyvalent (Fig 1D), which is typically 2–3 times greater than that of other similar products. Alternatively, immunisation strategies can substantially affect the efficacy of resulting antivenoms [29,30], and we encourage antivenom manufacturers to be more transparent with their immunisation strategies such that these factors can be carefully evaluated and thereby enabling their adoption by other manufacturers.

### Antivenom efficacy against viper venoms

Saw-scaled vipers (*Echis* spp.): Unsurprisingly, due to its medical and economic impact in West Africa [31], the efficacy of antivenoms against the venom of *Echis ocellatus* (west African saw-scaled viper) was investigated more than any other snake species (30 individual experiments using nine different antivenoms, Fig 1A). Antivenoms demonstrating preclinical efficacy against *E*. *ocellatus* venom exhibited ED_50_ values ranging from just 75.2 μl/mg to 2173.9 μl/mg, with a mean of 706.3 μl/mg (SD ±556.2 μl/mg) across all studies (Fig 2A, S1 Table). The least efficacious ED_50_ value reported for *E*. *ocellatus* (above) was determined using EchiTAbG (Micropharm, U.K.) [32], which also was reported in another study to have a potent preclinical efficacy of 80.8 μl/mg [33], an almost 27 fold difference. These studies were performed using different antivenom batches, in different laboratories and using venom sourced from distinct regions—Mali and Nigeria, respectively—and thus may account for the large differences in observed preclinical efficacy. However, the minimum preclinical efficacy of EchiTAbG appears sufficient for acceptable clinical efficacy based on positive outcomes from one of the very few antivenom clinical trials performed to date [34]. EchiTAb-Plus-ICP was tested eight times against *E*. *ocellatus* venom across five studies and provided more consistent mean ED_50_ values (ranging from 216.9 to 350.9 μl/mg); although these experiments were likely performed in the same laboratory (S1 Table). EchiTAb-Plus-ICP was also shown to be clinically effective in clinical trials against Nigerian *E*. *ocellatus* [34]. The ED_50_ of VINS Snake Venom Antiserum (Pan Africa) vs. *E*. *ocellatus* was examined once and determined to be greater than 4000 μl/mg, a value which the authors decided demonstrated a lack of preclinical efficacy against *E*. *ocellatus* venom [27]. Despite this, VINS Snake Venom Antiserum (Pan Africa) is currently indicated for use for treating envenoming by this species (Table 1). We are unable to determine if this particular product has been altered to improve efficacy since this 2016 report due to the lack of independent scrutiny. SAIMR Echis (South African Vaccine Producers, South Africa), an antivenom which has a long association with good treatment outcomes for African *Echis* envenoming [11,35,36], was examined preclinically twice against *E*. *ocellatus* venom from Nigeria, providing highly efficacious ED_50_s of 131.3 μl/mg [33] and 75.2 μl/mg [37]). Notably, SAIMR Polyvalent (South African Vaccine Producers, South Africa), an antivenom which is not indicated for the treatment of *E*. *ocellatus*, as this venom is not including in the immunising mixture, was found to have preclinical efficacy against *E*. *ocellatus* envenoming in the single study in which it was tested, although the resulting ED_50_ value was relatively lower compared to other products (934.1 μl/mg) [38]. The preclinical efficacy of the polyvalent-pan African Inoserp (Pan Africa) antivenom has been examined three times against *E*. *ocellatus* with higher than average ED_50_s of 1886.8 to 885.0 μl/mg (Fig 2A, S1 Table).

The efficacy of African antivenoms against the venom from other African species of saw-scaled viper (e.g. *E*. *leucogaster*, *E*. *pyramidum leakeyi*, *E*. *coloratus*) has been explored to a much lesser extent than *E*. *ocellatus* (Fig 1A). Five antivenoms exhibited broadly similar ED_50_ values against these *Echis* venoms as compared to *E*. *ocellatus* (S1 Table, Fig 2A), and included EchiTAbG (*E*. *coloratus* and *E*. *p*. *leakeyi* [4]), EchiTAb-Plus-ICP (*E*. *leucogaster* and *E*. *p*. *leakeyi* [39]), Fav-Afrique (Sanofi-Pasteur, [France]) (*E*. *leucogaster* [38]), SAIMR Echis (*E*. *p*. *leakeyi* [15]) and SAIMR Polyvalent (*E*. *leucogaster* [38]). The VACSERA antivenom was only tested against *E*. *coloratus* and *E*. *carinatus* venoms (presumably *E*. *p*. *leakeyi*, which was historically named *E*. *carinatus*) from Sudan, providing weakly efficacious ED_50_ values of 3333.3 and 2222.2 μl/mg, respectively [40].

African adders *(Bitis* spp.): The preclinical efficacy of antivenoms against venom from the puff adder (*Bitis arietans*), a large bodied medically important viper distributed widely across sub-Saharan Africa, was examined fourteen times in seven studies (Fig 1A). The ED_50_ values ranged enormously, from 3571.4 μl/mg (VACSERA) [40] to 14.9 μl/mg (SAIMR Polyvalent) [41](S1 Table). However, the majority of reported ED_50_s values (10/14) ranged from 393.0 to 215.5 μl/mg.

Few studies examined the preclinical efficacy of antivenoms against venoms from other medically important *Bitis* species, with only four antivenoms being tested, all of which were paraspecific to the venom in question (i.e. the venom-immunising mixture used to generate these antivenoms did not include venoms from these other *Bitis* species, or were not indicated for neutralisation of these venoms) (Fig 1A, Table 1). In general, and as perhaps expected, the resulting ED_50_ values demonstrated a greater quantity of these antivenoms are preclinically required against other *Bitis* species venoms as compared to *B*. *arietans* (range 395.0 to 1129.8 μl/mg for *B*. *gabonica*, 645.0 to 792.8 μl/mg for *B*. *rhinoceros*) (Fig 2A and S1 Table). Antivipmyn-Africa was reported as lacking preclinical efficacy against *B*. *nasicornis* venom [5] and EchiTAb-Plus-ICP also had poor efficacy (1639.0 μl/mg, S1 Table) against this venom [39]. No data were available for any of the four antivenoms that are marketed with specific claims of effectiveness against *B*. *gabonica* venom (VINS Snake Venom Antiserum [Pan Africa], VINS Snake Venom Antiserum [Central Africa], Fav-Afrique and Premium Snake Venom Antiserum [Pan Africa]) (Table 1).

### Antivenom efficacy against neurotoxic elapid venoms

Mambas (*Dendroaspis* spp.): Eight antivenoms were examined for their preclinical efficacy against *D*. *polylepis* (black mamba) venom (Fig 1A, Fig 2B). This snake is widely distributed throughout sub-Saharan Africa and its venom causes rapid systemic neurotoxicity. All tested antivenoms, with the exception of SAIMR-Polyvalent and EchiTAb-Plus-ICP, had ED_50_ values for *D*. *polylepis* ranging from 1030.9 to 5000.0 μl/mg. It is noticeable that these ED_50_ values are of considerably lower efficacy than those reported for the vipers described above (*E*. *ocellatus*, mean 706.3 μl/mg; *B*. *arietans*, mean 664.4 μl/mg). Of all antivenoms assessed, SAIMR Polyvalent exhibited the most efficacious ED_50_ values against *D*. *polylepis* venom (190.1–448.0 μl/mg), suggesting that this antivenom neutralises venoms from vipers and elapids with comparable efficacy (S1 Table, Fig 2B). EchiTAb-Plus-ICP, unsurprisingly, lacked efficacy in neutralising *D*. *polylepis* venom, as no mamba venoms are used in the immunising mixture[26].

Data on the preclinical efficacy of antivenoms against related green mamba species (genus *Dendroaspis*) were worryingly lacking (Fig 1A, Fig 2B). Four antivenoms were tested against *D*. *angusticeps*. Antivipmyn-Africa and VINS Snake Venom Antiserum (Central Africa) are not indicated for use against this species (Table 1) and neither possessed preclinical efficacy in neutralising venom-induced lethality at the doses tested (S1 Table)[5,25]. The VINS Snake Venom Antiserum (Pan Africa) and SAIMR Polyvalent antivenoms were examined in a single *D*. *angusticeps*-venom study and exhibited ED_50_s of 416.7 and 250.0 μl/mg, respectively. Preclinical efficacy of neutralisation of *D*. *viridis* venom was examined only once, where Antivipmyn-Africa exhibited an ED_50_ of 2850.0 μl/mg [5]; of substantial lower efficacy compared to other mamba venoms. We did not identify a single study reporting antivenom efficacy against *D*. *jamesoni* (of either subspecies) venom.

Cobras (*Naja* spp.): The other medically important neurotoxic elapids distributed across Africa are all members of the cobra genus *Naja*. With the exception of SAIMR Polyvalent, the majority of the antivenoms examined demonstrated generally weak (1111.1–5952.4 μl/mg) preclinical neutralising efficacies against neurotoxic cobra venoms (S1 Table).

Six antivenoms were tested for their preclinical efficacy against the venom of the snouted cobra (*N*. *annulifera*) (Fig 1A, Fig 2B). SAIMR Polyvalent, the only antivenom with specific claims for neutralising this venom, exhibited the most efficacious ED_50_ value (555.6 μl/mg, S1 Table)[26]—considerably lower ED_50_ values than obtained using the Antivipmyn-Africa and Fav-Afrique antivenoms (1700.1 and 1111.1 μl/mg, respectively)[5,26]. Three antivenoms (ASNA antivenom C, EchiTAb-Plus-ICP and Premium Snake Venom Antiserum [Pan Africa]) failed to demonstrate preclinical efficacy against the venom of *N*. *annulifera* at the doses tested (S1 Table)[26].

Despite its broad geographical distribution, only three antivenoms have been tested for their neutralisation of *N*. *haje* (Egyptian cobra) venom: VACSERA, SAIMR Polyvalent and Antivipmyn-Africa (Fig 1, Fig 2B). Although all three antivenoms prevented preclinical venom-induced lethality in mice, the ED_50_ values demonstrated poor efficacy (1742.3–5952.4 μl/mg) (Fig 2B, S1 Table)[5,15,40]. Similar ED_50_ values were reported for Antivipmyn-Africa, VACSERA and Fav-Afrique antivenoms against the venom of the forest cobra, *N*. *melanoleuca* (1778.8–2777.8 μl/mg) (Fig 2B, S1 Table)[5,38,40]. Two of three antivenoms (Antivipmyn-Africa and SAIMR Polyvalent) tested against *N*. *nivea* (cape cobra) venom, demonstrated efficacy in preventing venom-induced lethality in mice, although SAIMR Polyvalent was four times more efficacious (2115.2 vs. 529.9 μl/mg, respectively)[5,42] (Fig 2B, S1 Table). The VACSERA antivenom lacked preclinical efficacy in neutralising *N*. *nivea* venom at the doses tested[40] (S1 Table).

### Antivenom efficacy against cytotoxic elapid venoms

Cobras (*Naja* spp.) and rinkhals (*Hemachatus haemachatus*): Despite their medical importance[43,44], their widespread distribution[24,45] and the specific claims of ten products to be effective at neutralising envenoming of the predominately cytotoxic venoms of spitting cobras (Table 1), very few antivenoms have been scrutinised for their preclinical efficacy against the venoms of *Naja nigricollis*, *N*. *pallida*, *N*. *mossambica* or *N*. *nubiae*. Just four products (Antivipmyn-Africa, EchiTAb-Plus-ICP, SAIMR Polyvalent and VACSERA) have been tested against *N*. *nigricollis* venom (Fig 1A, Fig 2B, S1 Table) (although VINS Snake Venom Antiserum [Pan Africa], Inoserp Pan Africa and Premium Snake Venom Antiserum [Pan Africa] have been examined for their efficacy vs. *N*. *nigricollis* venom in a non-standard lethality assay[15]). EchiTAb-Plus-ICP was examined three times against *N*. *nigricollis* venom, resulting in ED_50_ values of 781.3, 2000.0 and 1428.6 μl/mg [24,46]. Antivipmyn-Africa and SAIMR Polyvalent demonstrated similar efficacies against this venom (1237.1 and 1133.9 μl/mg, respectively[5,15]). VACSERA demonstrated weak preclinical efficacies of 6060.6 and 3571.4 μl/mg against *N*. *nigricollis* venoms from Egypt and Sudan, respectively[40] (S1 Table). However, the former snake species may actually be *N*. *nubiae*, as the geographical distribution of *N*. *nigricollis* does not extend into Egypt.

The above four antivenoms had publicly available data on their preclinical efficacy against the venom of the East African spitting cobra species, *N*. *pallida* (Fig 1A, Fig 2B). Akin to the *N*. *nigricollis* results, Antivipmyn-Africa and SAIMR Polyvalent antivenoms exhibited similar ED_50_ values to one another (1761.9 and 1582.8 μl/mg, respectively[5,15]). EchiTAb-Plus-ICP (with *N*. *nigricollis* but not *N*. *pallida* in the venom-immunising mixture) demonstrated weaker efficacy (2631.6 μl/mg) against *N*. *pallida*[24] venom than against *N*. *nigricollis* venom (see above and Fig 1). The ED_50_ value of the VACSERA product against *N*. *pallida* venom (5494.5 μl/mg [40], S1 Table) was similar to that against *N*. *nigricollis* venom, above.

Reports of the preclinical efficacy of six antivenoms against the venom of the Mozambique spitting cobra (*N*. *mossambica*) yielded highly variable results (Fig 2B). Neither EchiTAb-Plus-ICP nor ASNA antivenom C antivenoms possessed efficacy in neutralising the preclinical lethal effects of *N*. *mossambica* venom from eSwatini (formerly Swaziland)[26] (S1 Table). However, EchiTAb-Plus-ICP did possess preclinical efficacy in neutralising *N*. *mossambica* venom sourced from Tanzania (1515.2 μl/mg)[26] (S1 Table, Fig 2B), while Antivipmyn-Africa had a preclinical neutralising efficacy of 1193.8 μl/mg [5] against *N*. *mossambica* venom of unknown origin. VACSERA possessed weak preclinical efficacy (2631.6 μl/mg) against *N*. *mossambica* venom similarly of unknown origin[40]. The efficacies of Fav-Afrique and SAIMR-Polyvalent antivenoms against *N*. *mossambica* venom were both of greater preclinical efficacy (both 714.3 μl/mg) than any of the other combinations of antivenom and spitting cobra venom[26].

Although not a *Naja* species, the rinkhals (*H*. *haemachathus*) is a closely related elapid snake species that spits venom, and envenoming exerts a predominately cytotoxic syndrome[47]. Four antivenoms (Antivipmyn-Africa, Premium Snake Venom Antiserum (Pan Africa), SAIMR Polyvalent and Fav-Afrique) were shown to possess preclinical efficacy against *H*. *haemachatus* venom (Fig 2B, S1 Table), with SAIMR Polyvalent, the only antivenom indicated for this venom in the immunising mixture (Table 1), being the most efficacious (ED_50_ 175.4 μl/mg). Antivipmyn-Africa, Premium Snake Venom Antiserum (Pan Africa) and Fav-Afrique possessed much lower efficacies of 1097.1, 1250.0 and 666.7 μl/mg, respectively[5,26]. Neither EchiTAb-Plus-ICP nor ASNA antivenom C possessed preclinical efficacy required to neutralise the lethal effects of *H*. *haemachatus* venom in mice (S1 Table)[26].

### The quality of *in vivo* parameter reporting

To assess the quality of reporting of *in vivo* ED_50_ experiments, we assessed the extent to which the data described in the 18 antivenom efficacy studies communicated the essential animal experimental parameters against 24 criteria guided by ARRIVE recommendations. We defined four areas of ED_50_ experiment reporting that would enable critical interpretation of results, protocol reproducibility and harmonisation of results from multiple reports: “Experimental setup”, “Animals”, “Procedure” and “Result reporting” (Table 2, S4 File). No single publication achieved the basic reporting requirements described under these criteria, and we found vast differences in reporting of the basic parameters of ED_50_ experiments (detailed in Table 2).

**Table 2:** Assessment of reporting standards for *in vivo* lethality antivenom assessment. ✓ = details reported. ✘ = details missing. F = female, M = male, B = both sexes, iv = intravenous injection, NP = non-parametric test, PB = Probit, SK = Spearman-Karber. Assessment criteria form basis of *in vivo* venom reporting checklist (S4 File).

Study reference	Experimental set up					Animals					Procedure								Result reporting				
	Antivenom batch	Total protein conc. of antivenom	Geographic origin of venoms	Ethical statement	Conflicts of interest statement	Source of animals	Mouse strain	Weight (g)	Mouse sex	Husbandry	Control groups stated	LD_50_ used for ED_50_	Number per group	Total number of animals usedgroups	Pre-incubation	Route of administration	Injection volume (ml)	Experiment length (h)	Group outcome reporting	Adverse events	Description of statistical analysis	ED_50_ calculation method	ED_50_ reporting
[5]	✓	✘	✘	✓	✓	✘	CD1	18–20	✘	✘	✓	3	6	✘	✓	iv	0.5	48	✘	✘	✓	NP	μl vs 3LD_50_
[39]	✓	✓	✓	✓	✘	✘	CD1	18–20	✘	✘	✓	5	5	✘	✓	iv	0.2	24	✘	✘	✘	SK	μl/mg
[27]	✓	✓	✓	✓	✓	✘	CD1	18–20	✘	✘	✘	5	6	✘	✓	iv	0.2	24	✘	✘	✓	PB	mg/ml
[32]	✓	✓	✓	✓	✓	✘	CD1	18–20	✘	✘	✓	5	5	✘	✓	iv	0.2	24	✘	✘	✓	PB	mg/ml
[46]	✓	✘	✓	✓	✓	✘	CD1	20–22	✘	✘	✓	3/ 5	6	✘	✓	iv	✘	48	✘	✘	✓	SK	mg/ml
[71]	✘	✓	✓	✓	✓	✘	CD1	20–22	✘	✘	✘	5	5	✘	✓	iv	0.2	✘	✘	✘	✘	✘	mg/ml
[37]	✘	✘	✓	✘	✓	✘	CD1	18–20	M	✘	✘	5	5	✘	✓	iv	✘	7	✘	✘	✘	PB	μg/mouse
[33]	✓	✓	✓	✓	✓	✘	✘	18–20	✘	✘	✘	5	✘	✘	✘	✘	✘	✘	✘	✘	✘	✘	μg/mouse
[4]	✘	✘	✓	✓	✘	✘	CD1	18–20	M	✘	✘	5	5	✘	✘	iv	0.2	7	✘	✘	✘	PB	μg/mouse
[38]	✘	✓	✓	✘	✘	✘	✘	✘	✘	✘	✘	3	5	✘	✓	iv	0.2	48	✘	✘	✘	SK	μg vs. 2LD_50_
[26]	✓	✓	✓	✓	✓	✘	CD1	18–20	✘	✘	✘	3/5	5	✘	✘	iv	0.2	24	✘	✘	✓	PB	mg/ml
[15]	✓	✓	✓	✓	✘	✘	CD1	18–20	M	✘	✘	2.5/ 5	5	✘	✘	iv	0.2	7	✘	✘	✘	PB	μl/mouse
[48]	✓	✘	✓	✓	✓	✓	CD1	18–20	B	✓	✓	4	4	✘	✓	iv	0.2	24	✘	✘	✘	PB	mg/ml
[24]	✓	✓	✓	✓	✓	✘	CD1	18–20	B	✘	✓	3	5	✘	✓	iv	0.2	48	✘	✘	✘	SK	μl/mg
[42]	✘	✘	✓	✓	✘	✓	CD1	18–20	M	✘	✘	2	5	✘	✓	iv	0.2	7	✘	✘	✘	PB	μl/mouse
[41]	✓	✘	✓	✘	✘	✘	BALB/c	18–20	F	✘	✓	3	8	✘	✓	iv	0.2	48	✘	✘	✘	SK	ml/mg
[25]	✓	✘	✓	✓	✓	✓	CD1	18–20	B	✘	✓	4	4	✘	✓	iv	0.2	24	✘	✘	✘	PB	mg/ml
[40]	✓	✘	✓	✘	✘	✘	Swiss	16–18	✘	✘	✘	✘	5	✘	✓	iv	0.5	24	✘	✘	✘	SK	LD_50_/ml

Experimental set up: Antivenom batches were reported by the majority (72%) of studies, as was the geographic origin of the venom(s) used to test antivenom efficacy (94%). Reporting of antivenom concentration, a key parameter when considering efficacy, was poor (Table 2). Half of the 18 studies did not provide the total protein concentration of the antivenoms tested, despite several reporting ED_50_s as milligrams of venom per milligram of antivenom (a compiled table of available total antivenom concentrations can be found in S2 Table). Where antivenom concentrations were reported, these ranged from 19 mg/ml to 171 mg/ml (Fig 1D, S3 Table).

Basic ethics statements were mentioned by most publications (78%), although only 11% met the level of transparency recommended by ARRIVE guidelines[20] (e.g. confirming compliance with all relevant ethical regulations and providing the name(s) of the board and institution that approved the study protocol). Conflict of interest statements were included for 61% of the studies (Table 2). Although we noted one instance of a study stating no conflict of interests despite employees of the antivenom manufacturer being authors on the manuscript[5].

Animals: Most manuscripts describing ED_50_ experiments reported the strain of mice used (88%), but two did not (Table 2). The majority of studies (77%) used the CD1 mouse strain, one study used BALB/c and another used Swiss albino. All but one article (94%) reported the weights of animals used (range 16-22g, majority 18-20g [14 of 18 studies]). The sex of the mice used was often not stated (66% failed to report), but for those that did we noted considerable variation, including the use of female, male or both sexes. Information on the husbandry of the mice was only reported in a single article[48].

Procedure: The LD_50_ value used to calculate the resulting ED_50_ potencies was reported by 94% of papers. The range of venom LD_50_ doses used to assess ED_50_ values ranged from 2x LD_50_ to 5x LD_50_, with the highest of this dose range the most frequently implemented (56% of studies, Table 2). Whilst the number of animals per group was routinely reported (range 4–6, mode = 5), the total number of groups or total number of animals used for experimentation was not reported by any study. Pre-incubation of venom-antivenom mixtures (78%), the route of injection (94%) and injection volumes (83%) were mostly well reported (although notable exceptions raise concerns over study reproducibility). The experiment length was reported by all but two papers, and was either 7 (22%), 24 (38%) or 48 (28%) hours (Table 2).

Result reporting: Eight different units for reporting ED_50_ values were presented (Table 2). The WHO recommended[10] units of: (i) mgs of venom neutralised per ml of antivenom (mg/ml), (ii) μl antivenom required to neutralise the “challenge dose” of venom (μl /mouse), or (iii) μl of antivenom required to neutralise 1 mg of venom (μl/mg), were used by 39%, 22% and 11% of publications, respectively. However, we note that the term “neutralised” in the WHO guidelines recommended definitions is ambiguous and possibly unhelpful, as it provides the impression that all venom is “neutralised” by the antivenom dose, which is not the case. A further five different approaches to define ED_50_ were used in 28% of the studies analysed here. The majority of publications reported on the statistical method used to calculate the ED_50_s - most commonly Probit (50%) or Spearman Kaber (33%)—although two studies did not. Standalone descriptions of *in vivo* statistical analysis were lacking in 72% of publications. The use of 95% confidence intervals alongside ED_50_ values were included in 89% of studies (Table 2). Finally, group outcome reporting was not provided in any study–this data is critical for future analysis and interpretation of results, especially in the case of extremely large confidence intervals. No paper reported the presence or absence of non-venom toxin mediated adverse effects/events in the experimental animals. Such adverse events do occasionally occur, particularly in the case of some antivenoms with high protein concentrations, where antivenom-venom mixtures produce observable precipitates (presumably the result of antibody/venom complexes) which can subsequently cause death in injected mice [15].

Finally, errors that can hamper interpretation of results (incorrect units and mislabelling in results tables) were identified in two manuscripts. We implore reviewers to pay extra attention to all ED_50_ values being reported, the methodology used, and to query dubious results.

## Discussion

Antivenoms are unusual human medicines because they are released for clinical use without seemingly needing to undergo the rigorous regulatory frameworks and expectations required for other drugs (e.g., clinical trials and transparent public access to data obtained regarding product efficacy [49,50]). This anomaly likely reflects historical and prevailing lack of national and international investment in snakebite management and regulatory frameworks pertaining to serotherapies. As a consequence, results from murine preclinical efficacy assays have become the primary means to assess/predict antivenom performance. However, because the tropical countries that most need antivenom rarely possess the requisite antivenom preclinical testing facilities, and because of the lack of independent tests, an over-reliance upon manufacturer’s product claims has evolved. In this study we (1) evidence and identify the risks posed by this situation and suggest remedial actions, (2) describe how the data provided by 18 carefully-selected publications of preclinical antivenom efficacy testing can be useful to decision making by clinical, medicines-regulatory agency, manufacturing and academic stakeholders, and (3) call for greater investment in preclinical and clinical testing of antivenoms before they are released for human use.

### 1. The risks inherent to the variation in current antivenom preclinical testing protocols and their reporting

#### The lack of publicly available data

There is no requirement for antivenom manufacturers to publish their preclinical testing results. The expertise to rigorously scrutinise this data can be lacking in the drug regulatory agencies of resource-poor countries that most need effective antivenom. Similarly, clinicians treating snakebite patients, especially those without snakebite management experience, can struggle to use the documentation accompanying a vial of antivenom to make confident/accurate clinical decisions [15,51]. Academic groups have tried to fill this knowledge gap. This study, however, has revealed a disturbing lack of published data of the preclinical efficacy for antivenom products used in sub-Saharan Africa in the last 20 years. Notable gaps include:

- published data for the majority of polyspecific African antivenoms is only available for a subset of the venoms that they claim to neutralise (Fig 1A)
- there exists almost no publicly available reports on the preclinical efficacy or clinical effectiveness [11] for some of the more affordable and widely-available antivenoms [15,51].
- the most comprehensive antivenom preclinical testing reports (wherein efficacy against a large variety of venoms were examined) emanated from standalone studies which involved the antivenom manufacturer [5,40]

While neutralisation of venoms from most of the medically important snakes of Africa (e.g., *E*. *ocellatus*, *D*. *polylepis*) have been tested for more than one antivenom and on more than one occasion (Fig 1A), we were unable to find a single preclinical testing publication for many WHO category 1 (highest medical importance) species including *D*. *jamesoni*, *N*. *anchietae*, *N*. *ashei*, *N*. *katiensis*, *N*. *nigricincta*, *N*. *senegalensis* and *E*. *jogeri*.

These knowledge-gap examples highlight the urgent need for national and international communities to (i) invest in the delivery of thorough preclinical testing of antivenom products and (ii) ensure the results of these studies are made publicly available. These gaps in preclinical data need to be addressed urgently to avoid the clinical use of inappropriate antivenoms that have led to dramatic increases in case fatality rates[12,13] and to inform antivenom clinical trial designs[3].

#### The quality of preclinical testing data

The variant ED_50_ protocols and efficacy metrics employed by different laboratories in their publications (Table 2) limits opportunities for meta analyses. This is further compounded by some laboratories using the intraperitoneal route of injection to assess antivenom efficacy (which have not been considered here). Furthermore, the ongoing reproducibility crisis in animal research [52] undoubtedly applies to preclinical antivenom efficacy research. This is especially important given the current reliance on this data for approving antivenoms for human use [10]. The examples below evidence some of these inconsistencies and their potential impact:

i Differences in venom LD_50_ values used to determine antivenom ED_50_s values for the same species:
  - The reports of ED_50_ values for *E*. *ocellatus* venom-neutralisation used 2 [38], 3 [5] and 5 (all remaining studies) times the venom LD_50_ dose. The ED_50_s for *B*. *arietans* were reported using 3 [5,41] and 5 (majority of studies) times the venom LD_50_ doses, or not stated at all [40]. The majority of ED_50_ values reported for Antivipmyn-Africa were obtained using 3 x LD_50_s [5] (S1 Table).
  - This is important, as the higher the challenge dose, the lower the estimation of preclinical efficacy [18,53]. For example, Bogarin et al.[54] demonstrated that ED_50_ values of 163.9, 310.6 and 500.0 μl/mg were obtained for the same antivenom when tested against 3, 4 or 5 *Bothrops asper* venom LD_50_s, respectively.

Insufficient consideration of the venom challenge dose used in different effective median dose experiments can mask important information on an antivenom’s efficacy. We therefore recommend an international, species-specific consensus is agreed on the number of venom LD_50_s used for measuring preclinical antivenom efficacy. For sub-Saharan African venoms, we propose a 5 x venom LD_50_ dose in the majority of cases, with exemptions where high venom LD_50_s values limit the amount of antivenom that can be administered [15].

ii Differences in ED_50_ metrics and their confidence intervals:
  - Eight different preclinical efficacy metrics were used across the 18 studies investigated here (Table 2). To undertake the cross-antivenom analysis reported here, we needed to convert all the individual metrics into the μl/mg metric.
  - Some studies reported ED_50_s with very large confidence intervals (Fig 2, S1 Table). For example, for SAIMR Polyvalent against *B*. *arietans*, the sample mean ED_50_ in one study was given as 373.2 μl/mg [37], however, based on the 95% confidence intervals (106.1–561.6 μl/mg), the true mean efficacy could be as low as 106.1 μl/mg. Thus, this antivenom might be 3.5-fold more potent than described. Conversely, the ED_50_ of ASNA antivenom C for neutralising *B*. *arietans* venom was reported as 263.2 μl/mg [26], but the 95% confidence intervals (200.0–666.7 μl/mg) suggest the population mean could be as high as 666.7 μl/mg, and thus the product may be more than 50% less potent than suggested. While large confidence intervals are likely partially unavoidable due to the complex pathophysiology of envenoming (where death often results from a multitude of cardiovascular and/or haemostatic effects, such as shock or intercranial haemorrhage[55]), they may also be due to attempting to calculate ED_50_ values with insufficient data (through use of insufficient animals (<4) per, or number of, groups). The resulting high range of some confidence intervals risks inaccurate estimation of an antivenom’s preclinical efficacy.

This situation is clearly far from desirable, for all the reasons stated above, and especially because of the potential over-estimation or under-estimation of an antivenom’s efficacy. Whilst the WHO guidelines for antivenom preclinical efficacy testing suggest three metric options [10], we argue that it is only through the standard use of a single metric that these reports can be useful to clinicians, clinical trial designers and regulatory agencies–and we propose the use of the dose of antivenom that protects 50% of mice per mg of venom (μl/mg) in all reports (with use of other metrics as secondary descriptors left to the authors’ discretion). Whilst not the most frequently used metric for describing antivenom efficacy (11% of the identified studies) (Table 2), our reasons for selecting this particular metric are: i) it is easily understandable for people unfamiliar with the assay to identify antivenoms that possess greater efficacy without the requirement for additional information, and ii) it is within keeping of the pharmacological definition of effective dose (e.g. a dose or concentration of a drug that produces a defined response).

The original intention of Theakston and Reid’s (1983) standardised preclinical test of antivenom efficacy [17] was to enable antivenoms to be “graded easily and uniformly, in terms of their venom neutralising abilities, by different laboratories”–this is currently not being achieved. To help facilitate standardisation of reporting of preclinical antivenom efficacy experiments, we have developed an experimental reporting checklist (S4 File), guided by ARRIVE recommendations, primarily for use in publishing antivenom preclinical testing in academic journals. The criteria therein are the same as we used to assess the publications in this paper (Table 2). We welcome ideas and suggestions to improve this document, and once agreement has been reached with others in the community, we recommend that it be added to the WHO guidelines for the production, control and regulation of snake antivenom immunoglobulins.

We estimate that 3,930 mice were used to generate the results of the 18 publications examined here (excluding mice required to estimate venom LD_50_s doses). Whilst ‘cost-to-mice/benefit-to-human health’ considerations undeniably justify these murine experiments, especially in the absence of routine clinical trials, these assays are severe in nature and are distressing for animals and researchers alike. In-line with recommendation in the WHO guidelines [10], we urge the adoption of refined antivenom preclinical testing protocols, such as: the use of analgesia [56], dose staging [15], humane endpoints [23] and reduced experimental time [4], after a period of careful validation.

#### Issues posed by the geographical origin of venoms used for preclinical testing

Venom content can vary substantially from region to region [8]. For example, the protein composition of *B*. *arietans* venoms are known to exhibit geographical variation [6], and this species may well represent a species complex. Similarly, *N*. *melanoleuca* was recently taxonomically reclassified into five species[57], with unknown implications regarding venom protein compositions. However, very few antivenoms have been preclinically tested on venoms from the same species sourced from different geographical locales (Fig 1B, Fig 2, S1 Table). The notable exceptions being EchiTAbG and EchiTAb-Plus-ICP for *E*. *ocellatus* and SAIMR Polyvalent for *B*. *arietan*s and, to a lesser extent, *D*. *polylepis*. We therefore advise against assuming that the preclinical efficacy of an antivenom against a venom from one region can be simply extrapolated to other regions. The dangers of such assumptions are apparent from the variable preclinical efficacy of Russell’s viper (*Daboia russelii*) antivenoms manufactured with venom sourced from India against venoms from Bangladesh, Pakistan and Sri Lanka [7].

Our analysis illustrates that venoms used for preclinical testing have tended to be sourced from regional “hot spots” (Fig 1B). This results in an absence of antivenom efficacy data for large swathes of sub-Saharan Africa, particularly central and southern countries suspected of having substantial snakebite burdens [58,59]. It is therefore important that the scientific community expands preclinical testing of antivenoms to include venoms from different medically-relevant regions. We also implore antivenom manufacturers to be more transparent on the origins of the venoms used in their immunisation procedures and subsequent assessments of preclinical efficacy [19].

### 2. Use of the data provided by the 18 selected publications to inform decision making

The very limited data on the clinical effectiveness of African antivenoms [3,11,60] has enforced a reliance upon preclinical efficacy data to assess an antivenom’s potential clinical utility. The results presented in Fig 2A identify that several of the antivenoms possess comparable preclinical efficacy against *B*. *arietans* (puff adder) venom. The five studies reporting the highest efficacy against *E*. *ocellatus* (saw-scaled viper) venom included the *Echis* monovalent, SAIMR Echis and EchiTAbG antivenoms and the trivalent EchiTAb-Plus-ICP antivenom. Several antivenoms exhibited comparable preclinical efficacy against the lethal effects of a variety of spitting cobra venoms, but the clinical value of this is questionable because human envenoming by these species rarely results in fatalities (see below). The SAIMR Polyvalent antivenom showed markedly and consistently higher preclinical efficacy against venoms of the neurotoxic cobras (*N*. *haje*, *N*. *melanoleuca*. *N*. *nivea*) and the black and green mambas (*D*. *polylepis*, *D*. *angusticeps*) than any other single product.

Based on very limited human clinical evidence [11], African antivenoms with high ED_50_ doses should be treated with caution when considering their ability to neutralise envenoming clinically. For example, Antivipmyn-Africa demonstrates ED_50_s of 1694.9–2850.0 μl/mg against neurotoxic elapids (Table 1), and thus this antivenom is ‘efficacious’ preclinically, albeit with low efficacy. However, very limited investigations into this antivenom’s clinical effectiveness have suggested it works poorly against *D*. *polylepis* envenoming (Antivipmyn-Africa vs. *D*. *polylepis* ED_50_ = 1694.9 μl/mg) [11,61]. While we must extrapolate with caution, given that many antivenoms possess similarly high ED_50_ dose values as Antivipmyn-Africa against neurotoxic venoms (S1 Table, Fig 2), any potential clinical effectiveness of such products against neurotoxic snake venoms should be carefully evaluated before advocating for their widespread use.

Systemic life-threatening envenoming is not the only consequence of snakebite. Many more victims per year suffer from the debilitating local effects of snake venoms. Thus, the continued marketing of antivenoms solely on the basis of their efficacy in neutralise systemic lethal effects of envenoming is an ongoing concern [18]. Envenoming by cytotoxic elapids, such as *N*. *nigricollis*, *N*. *pallida* and *N*. *mossambica*, primarily inflict substantial local necrosis [62–64] and not the lethal systemic neurotoxic effects that are the primary measure of ED_50_ testing of elapid venoms [62]. Additionally, vipers such as the *Bitis* and *Echis* genera cause extensive local necrosis in addition to systemic pathologies [55,62]. As recommended by the WHO [10], preclinical examination of antivenom using the Minimum Necrotizing Dose (MND) assay is required *in addition to* the ED_50_ assay to provide a more comprehensive analysis of an antivenom’s efficacy. In this study, of the 16 papers detailing antivenom efficacies against venoms inflicting local pathology (S1 Table), just two articles included results of MND efficacies [24,39]. Thus, in the absence of evidence of preclinical efficacy of an antivenom in the MND assay, alongside the total absence of clinical trials for any antivenom against African spiting cobras, ED_50_ values should be considered with extreme caution when interpreting their use for treating envenomation from African spitting cobras.

#### The challenge of extrapolating antivenom dose from preclinical data

There is a lack of international consensus on the acceptable limits for the preclinical efficacies of antivenoms other than the concept that they must prevent the most relevant toxic activities. An antivenom that is unable to preclinically prevent venom-induced lethality *in vivo* should clearly not be accepted for clinical use. However, as evidenced by the example of Antivipmyn-Africa vs. *D*. *polylepis*, above [11,61], just because an antivenom can preclinically neutralise venom toxicity in a murine model (e.g. at any ED_50_ value), does not mean that this product will be an effective snakebite therapy in clinical settings. An antivenom must not be labelled as preclinically efficacious just because it can neutralise a venom at any ED_50_ value. Several reported ED_50_ values collated in this study are poor and fall short of some of the preclinical efficacy cut off thresholds employed by different researchers [5,24–26]. Whilst the use of such minimum ED_50_ efficacy thresholds is controversial[19], common sense should prevail.

Furthermore, ED_50_ values need to be interpreted on a species-specific basis. Similar ED_50_ values from the same antivenom against two different snake species does not mean that the antivenom will be equally effective against bites by both species in clinical settings. The ED_50_ potency is ultimately determined against a challenge dose of venom based on the species-specific LD_50_ value, thus the quantity of venom required to achieve a challenge dose varies substantially between species and within species from different locations (S2 Table). Perhaps most importantly, these analyses do not consider the amount of venom which can be delivered during a snake bite, which can vary from less than 10 mg (e.g. mean venom yields of *E*. *p*. *leakeyi* and *E*. *ocellatus* reported as 7.4[±2.6] and 10.2[±4.9] mg) to routinely greater than 150 mg of venom (e.g. mean yields of *B*. *arietans* and *N*. *nigricollis* reported as 166[±76.0] and 159.7[±86.2] mg, respectively)[65].

Ultimately, the final proof of antivenom effectiveness and safety must be shown in the clinical setting. Preclinical data can be used to guide the selection of a tentative dose for clinical use which can then be tested in clinical trials, as previously successfully attempted for a monospecific and trispecific antivenom [33]. However, it is worth stressing that predicting appropriate therapeutic doses of antivenom for use in snakebite victims from preclinical efficacy ED_50_ data is exceedingly problematic. This is due to the fact that in the standard ED_50_ preclinical assay, venom is delivered intravenously, a scenario which happens rarely during a snakebite, and already pre-incubated with antivenom, which does not happen in the real-world. Thus, while these experiments remain useful for assessing the preclinical efficacy of an antivenom, and for providing potency comparisons between different products, the model remains exceedingly limited in terms of representing a ‘real-world’ envenomation scenario. For this reason, in recent years, a number of groups have moved towards the use of “rescue” experiments, where venom is often delivered by different routes and treatment is administered after the onset of envenomation [66–69]. However, it is clear that to use preclinical data to more rationally and precisely model the therapeutic doses required to effect cure, a more comprehensive understanding of the following is needed; i) the relative amount of toxin-neutralising antibodies in antivenom products, such as provided by antivenomic analyses [16], ii) the amount and type of venom toxins circulating in snakebite victims over the time course of an envenoming and in different tissue compartments and iii) the concentrations of antivenom required to bind and inhibit the pathogenicity of venom toxins in that time frame and tissue compartment. Such pharmacodynamic data is routine for the majority of licensed therapeutics that have successfully progressed through normal clinical-trial processes[70]. Even with the advancement of antivenom preclinical testing to more closely match real-life envenoming, all antivenoms must ultimately still be tested in clinical trials.

### 3. Greater investment in preclinical testing of antivenoms is required to maximise their utility to all stakeholders

This analysis of the variation in the reports of, and protocols used for, preclinical antivenom efficacy testing in sub-Saharan Africa has identified several issues that can be readily remedied, including:

- Publication of results in formats and journals that maximise access
- Adherence to standard protocols for assessing venom LD_50_ and antivenom ED_50_ values
- Standardisation of LD_50_ challenge doses
- Utilisation of a single metric for reporting antivenom ED_50_ values–we recommend μl/mg
- Transparent, detailed reporting of preclinical outcomes to allow independent analysis, interpretation and robust peer-review of antivenom ED_50_ values
- Agreement on acceptable 95% CI ranges for antivenoms
- Transparent reporting of antivenom concentration, experiment duration (hours), venoms used for immunisation–especially their precise country of origin, and other protocols

The simplest way for these beneficial changes to be implemented would be for the WHO and relevant journals to adopt and encourage use of a reporting checklist, like the one presented here (S4 File). Failing that, the WHO guidelines should at a minimum promote the use of reporting antivenom preclinical efficacy in academic journals using the ARRIVE guidelines, whilst relevant journals should make concerted efforts in enforcing its use for reporting such studies.

Perhaps the most important outcome of this analysis is the very obvious and urgent need for more rigorous and comprehensive preclinical testing of antivenoms designed for sub-Saharan Africa. These need to encompass the above points and include a diversity of medically-relevant venoms from geographically diverse countries. Achieving this will require significant investment from national and international agencies. It will also need a strategy to prevent breaches of the Nagoya protocol, which forbids the unprotected movement of biological material from its country of origin. The most effective, capacity strengthening (but expensive) solution is the establishment of manufacturer-independent antivenom preclinical testing units in key locations throughout sub-Saharan Africa. These would provide regional venom resources, prevent use of antivenoms of questionable efficacy and provide the expertise and knowledge needed for regional antivenom clinical trials. Investment in this infrastructure will have lasting benefits to human health.

## Supporting information

S1 Table List of all ED_50_ values reported in identified studies. (XLSX)

S2 Table List of total protein contents of antivenoms investigated in this study. (XLSX)

S3 Table List of all LD_50_ values reported in identified studies. (XLSX)

S1 File List of literature search terms. (DOCX)

S2 File Details of all publications returned by literature search. (XLSX)

S3 File Details of calculations for conversions of ED_50_ and potency values. (XLSX)

S4 File *In vivo* venom experimental reporting guidelines. (DOCX)
