# Supplementary material for: An analysis of preclinical efficacy testing of antivenoms for sub-Saharan Africa: Inadequate independent scrutiny and poor-quality reporting are barriers to improving snakebite treatment and management
Source: PLoS Negl Trop Dis. 2020 Aug 20;14(8):e0008579. doi: 10.1371/journal.pntd.0008579 (PMC7462309; doi:10.1371/journal.pntd.0008579)
Supplement: S1 File — (DOCX) [file pntd.0008579.s004.docx]

**Supplementary File 1: List of search terms used for literature search.**

Initial search performed on 2-8-19. Subsequent search performed on 11^th^ of May (note – this search did not include Scopus due to no longer having access)

search terms used:

antiven* OR Anti-snake* OR ASV OR Anti-ophidic AND africa

antiven* OR Anti-snake* OR ASV OR Anti-ophidic* AND africa AND preclinical

antiven* OR Anti-snake* OR ASV OR Anti-ophidic* AND africa AND Effective Dose

antiven* OR Anti-snake* OR ASV OR Anti-ophidic* AND africa AND Lethal Dose

antiven* OR Anti-snake* OR ASV OR Anti-ophidic* AND africa AND Echitab*

antiven* OR Anti-snake* OR ASV OR Anti-ophidic* AND africa AND SAIMR

antiven* OR Anti-snake* OR ASV OR Anti-ophidic* AND africa AND Premium

antiven* OR Anti-snake* OR ASV OR Anti-ophidic* AND africa AND VINS

antiven* OR Anti-snake* OR ASV OR Anti-ophidic* AND africa AND VACSERA

antiven* OR Anti-snake* OR ASV OR Anti-ophidic* AND africa AND Inoserp

antiven* OR Anti-snake* OR ASV OR Anti-ophidic* AND africa AND ASNA

antiven* OR Anti-snake* OR ASV OR Anti-ophidic* AND africa AND Antivipmyn

antiven* OR Anti-snake* OR ASV OR Anti-ophidic* AND africa AND Fav-afrique

Searched: Scopus, Pubmed and Web of Science

Limited to publications from 1999 (inc.) onwards.

Limited to publications in English, French, Portuguese and Spanish.
