# Supplementary material for: An analysis of preclinical efficacy testing of antivenoms for sub-Saharan Africa: Inadequate independent scrutiny and poor-quality reporting are barriers to improving snakebite treatment and management
Source: PLoS Negl Trop Dis. 2020 Aug 20;14(8):e0008579. doi: 10.1371/journal.pntd.0008579 (PMC7462309; doi:10.1371/journal.pntd.0008579)
Supplement: S4 File — (DOCX) [file pntd.0008579.s007.docx]

***In vivo* venom experiment reporting checklist .**

This checklist has been written in relation to the ARRIVE guidelines^1^ for increased reproducibility in animal experimentation ([available here](https://www.nc3rs.org.uk/arrive-guidelines)). The checklist is specifically designed so that the minimal information required for accurate reporting and reproducibility of *in vivo* antivenom efficacy experiments can be provided in all future publications. Whilst specifically written for ED_50_ assays, many areas of this checklist are applicable to all *in vivo* murine venom experiments.

Ideally, this checklist should be referred to **before** the onset of *in vivo* experimentation, to ensure the required information is accurately recorded. Detailed WHO guidelines on procedures for performing antivenom preclinical efficacy testing can be found in Annex 5 of the *WHO Guidelines for the Production, Control and Regulation of Snake Antivenom Immunoglobulins*^2^ ([available here](https://www.who.int/bloodproducts/snake_antivenoms/snakeantivenomguide/en/)).

**> Prior to *in vivo* experimentation**________________________________.

Prior to any *in vivo* preclinical analyses, comprehensive research should be undertaken which a) justifies use of animals and b) ensures unproductive or redundant use of animals is avoided. Details of this research should be included in all publications.

Examples of such research primarily consist of:

1. Literature searches to ensure experiments have not already been performed and desired data (e.g. LD_50_ values) are not already available.
2. Comprehensive *in vitro* analyses (such as ELISA, immunoblotting, antivenomics analyses and various functional assays) which will deselect any potential therapy which is unlikely to be efficacious *in vivo.*

**> Experimental set up**___________________________________________.

Detail venom(s) used, including:

Supplier

Geographical origin of the specimens providing the venom.

*Example: “D. typus venom was obtained from Latoxan SAS, Valence, France. The venom is a pool obtained from several specimens collected in Tanzania.”*

Details of antivenom(s) used, including:

Batch

Expiry date

*Example:* “*The following polyspecific antivenoms were used: (a) SAIMR (South African Institute for Medical Research) Polyvalent Snake Antivenom from South African Vaccine Producers (Pty) Ltd. (batch number BC02645, expiry date 07/2016); (b) Snake Venom Antivenom (Central Africa) from VINS Bioproducts Ltd. (batch 12AS13002, expiry date 04/2017); and (c) Snake Venom Antivenom (African) from VINS Bioproducts Ltd. (batch 13022, expiry date 01/2018)*^3^*.”*

Antivenom total protein content (in mg/ml) and the method by which it was determined

Ethics:

Ethical compliance

Provide a statement confirming that you have complied with all relevant ethical regulations and that that you have stated the name(s) of the board and institution that approved the study protocol.

Conflict of interest statement

These should follow individual journal guidelines. If a journal does not have a policy on conflicts of interest, please refer to: <https://www.nature.com/nature-research/editorial-policies/competing-interests>. A common conflict on interest in the snakebite envenomation field is the association of journal authors with antivenom manufacturers (frequently as employees or consultants). Any such associations should be clearly declared.

**> Animals**  ______________________________________________.

The following should be listed in all *in vivo* reporting:

Source of animals

Mouse strain

Mouse weight

Sex

Basic husbandry details

*Example: “Male CD1 mice (18-20g) were purchased from Charles River, U.K. Mice were kept in specific pathogen free conditions, with water and food ad libitum.”*

**> Procedure .**

Control groups stated (if applicable)

It is ethically questionable whether it is necessary to always perform control experiments (e.g. venom only) each time a neutralisation of lethality experiment is performed. If the venom batch being used has already been validated, then there is no need to unproductively use a further group of mice to repeat the result. PBS only control groups add little, if any, value and are discouraged.

Number of mice per group

Total number of animals and number of groups used

*Example: “For each of the 4 venoms, 5 groups (consisting of 5 mice/group) received venom-antivenom mixtures at varying antivenom concentrations (total 100 mice, see table X for a detailed description of individual dosing groups)”*

Numbers of LD_50_ used for ED_50_ (ideally also expressed in µg/mouse or mg/kg)

If LD_50_s are not calculated during the current experimental study, a reference must be supplied to the study describing the LD_50_ dose used. Note: if using less than 2.5 LD_50_ doses, a justification (e.g. constrained by maximum i.v. dose volume) should be supplied.

If and how venom was pre-incubated with venom

Route of administration

Injection volume

Experiment length (in hours)

*Example: “Varying doses of antivenom were mixed with 5 LD_50_s and incubated for 30 minutes at 37 °C using a water-bath. Mixtures were then kept on ice until administration. Venom-antivenom mixtures were administered in volumes of 200 µl via the tail vein. Numbers of deaths/survivors at 24 hours were recorded.”*

Note, **in detail**, additional refinements to the experiment, if applicable

This could be the use of analgesia (brand, dose, route, dosing regimen)^4^, implementation of humane endpoints^5^ or increased frequency of monitoring^6^.

**>Result reporting .**

Essential:

Group outcome reporting

This should be supplied as a supplemental table. All antivenom doses used and the numbers of deaths/survivors per group should be recorded.

The presence or absence of adverse events

e.g. misinjection, toxin-antibody precipitates or antivenom-related adverse reactions.

Description of *in vivo* statistical analysis

As a minimum, the technique of how ED_50_ values were calculated (either by Probits, Spearman-Karber or by non-parametric methods) should be clearly stated. Additional statistical analysis should be clearly described in the materials and methods.

Standardised units for ED_50_ reporting

ED_50_s should be reported as the dose of antivenom, in microlitres, which prevents lethality in 50% of test mice per milligram of venom (µl/mg) alongside 95% confidence intervals. Other WHO recommended units (mg of venom neutralised^[[1]](#footnote-1)^ by one ml of antivenom [mg/ml] or dose of antivenom which neutralises the “challenge dose” of venom µl/µg ), whilst perhaps useful for venom researchers, are of little use to clinicians or national regulatory agencies and hamper efforts at comparing antivenom pre-clinical efficacy. If desired, these units can be reported *in addition to* µl/mg if the author feels it is necessary. Since LD_50_ values for the same venom may vary from one supplier/geographical region to another, reporting of ED_50_ as the number of murine LD_50_s of venom neutralised per ml of antivenom is not permitted.

Optional:

Whilst “optional” these observations and metrics allow much more information to be generated from a single ED_50_ experiment and will allow further refinement of procedures for laboratories globally.

Time of death

Measured in minutes or hours from the onset of envenomation^5,6^.

Characteristics of typical envenoming progression

e.g. paralysis, seizure, bleeding from injection site. Whilst this may seem obvious, these observations are very useful for laboratories performing venom experiments with little/no prior experience

Details of any unexpected observations of envenoming

e.g. hyperactivity, initial slump with recovery, unexpected paralysis, blindness

Details of macroscopic post-mortems

e.g. extent/location of internal haemorrhage, myonecrosis, anaemia, etc

**>Definitions of key terms**

*Median lethal dose* (LD_50_)*:* the quantity of snake venom that leads to the death of 50% of the animals in a group after an established period of time ^2^.

*Median effective dose* (ED_50_)*:* the quantity of antivenom that protects 50% of test animals injected with a lethal dose (typically 2.5-5 x LD_50_) of venom ^2^*.* Alternatively, this can be viewed as the quantity of antivenom that neutralises all but 1 x LD_50_.

*Efficacy*: the efficacy of an antivenom is a measure of the *in vivo* or *in vitro* neutralising potency against a specific activity of a venom or venoms ^2^.

*Effectiveness*: the effectiveness of an antivenom is a measure of its ability to produce a clinically effective outcome when used to treat snakebite envenoming ^2^.

*Antivenom Potency:* This unit considers the challenge dose of venom and thus refers to the ratio of milligrams of venom neutralised per mL of antivenom in which there is 100% protection (E.g. ED_100_). See ^7,8^ for examples.

**>References**

1. Kilkenny, C., Browne, W. J., Cuthill, I. C., Emerson, M. & Altman, D. G. The ARRIVE. **8**, 6–10 (2010).

2. World Health Organization (WHO). Guidelines for the production, control and regulation of snake antivenom immunoglobulins. *WHO* (2018).

3. Laustsen, A. H., Lomonte, B., Lohse, B., Fernández, J. & Gutiérrez, J. M. Unveiling the nature of black mamba (Dendroaspis polylepis) venom through venomics and antivenom immunoprofiling: Identification of key toxin targets for antivenom development. *J. Proteomics* **119**, 126–142 (2015).

4. Herrera, C., Bolton, F., Arias, A. S., Harrison, R. A. & Gutiérrez, J. M. Analgesic effect of morphine and tramadol in standard toxicity assays in mice injected with venom of the snake Bothrops asper. *Toxicon* **154**, 35–41 (2018).

5. Ainsworth, S. *et al.* The paraspecific neutralisation of snake venom induced coagulopathy by antivenoms. *Commun. Biol.* (2018). doi:10.1038/s42003-018-0039-1

6. Albulescu, L.-O. *et al.* A Decoy-Receptor Approach Using Nicotinic Acetylcholine Receptor Mimics Reveals Their Potential as Novel Therapeutics Against Neurotoxic Snakebite. *Front. Pharmacol.* (2019). doi:10.3389/fphar.2019.00848

7. Morais, V., Ifran, S., Berasain, P. & Massaldi, H. Antivenoms: Potency or median effective dose, which to use? *Journal of Venomous Animals and Toxins Including Tropical Diseases* (2010). doi:10.1590/S1678-91992010000200002

8. Pla, D. *et al.* Phylovenomics of Daboia russelii across the Indian subcontinent. Bioactivities and comparative in vivo neutralization and in vitro third-generation antivenomics of antivenoms against venoms from India, Bangladesh and Sri Lanka. *J. Proteomics* (2019). doi:10.1016/j.jprot.2019.103443

1. “Neutralisation” is an ambiguous term which can lead to substantial confusion in the context of ED_50_ experiments. In ED_50_ experiments, challenge doses are not completely neutralised, as 50% of the mice still succumb to lethal venom effects. We suggest avoiding statements like “The Antivenom demonstrated a *neutralising* efficacy of 700 µl/mg" and instead consider describing efficacies as “The antivenom had a *preclinical* efficacy of 700 µl/mg”. [↑](#footnote-ref-1)
